# Characterization and mechanism of the effects of Mg–Fe layered double hydroxide nanoparticles on a marine bacterium: new insights from genomic and transcriptional analyses

**DOI:** 10.1186/s13068-019-1528-2

**Published:** 2019-08-16

**Authors:** Wei Ren, Yanshuai Ding, Lide Gu, Wanli Yan, Cang Wang, Mingsheng Lyu, Changhai Wang, Shujun Wang

**Affiliations:** 1 Jiangsu Key Laboratory of Marine Bioresources and Environment/Jiangsu Key Laboratory of Marine Biotechnology, Jiangsu Ocean University, Lianyungang, 222005 Jiangsu People’s Republic of China; 2 Jiangsu Provincial Key Laboratory of Marine Biology, College of Resources and Environmental Sciences, Nanjing Agricultural University, Nanjing, 210095 Jiangsu People’s Republic of China; 3 Collaborative Innovation Center of Modern Bio-manufacture, Anhui University, Hefei, 230039 Anhui People’s Republic of China; 4 Co-Innovation Center of Jiangsu Marine Bio-industry Technology, Jiangsu Ocean University, Lianyungang, 222005 Jiangsu People’s Republic of China

**Keywords:** Marine bacteria, *Arthrobacter oxidans* KQ11, Mg–Fe layered double hydroxide nanoparticles, Dextranase, Transcriptional profiling

## Abstract

**Background:**

Layered double hydroxides (LDHs) have received widespread attention for their potential applications in catalysis, polymer nanocomposites, pharmaceuticals, and sensors. Here, the mechanism underlying the physiological effects of Mg–Fe layered double hydroxide nanoparticles on the marine bacterial species *Arthrobacter oxidans* KQ11 was investigated.

**Results:**

Increased yields of marine dextranase (Aodex) were obtained by exposing *A. oxidans* KQ11 to Mg–Fe layered double hydroxide nanoparticles (Mg–Fe-LDH NPs). Furthermore, the potential effects of Mg–Fe-LDH NPs on bacterial growth and Aodex production were preliminarily investigated. *A. oxidans* KQ11 growth was not affected by exposure to the Mg–Fe-LDH NPs. In contrast, a U-shaped trend of Aodex production was observed after exposure to NPs at a concentration of 10 μg/L–100 mg/L, which was due to competition between Mg–Fe-LDH NP adsorption on Aodex and the promotion of Aodex expression by the NPs. The mechanism underling the effects of Mg–Fe-LDH NPs on *A. oxidans* KQ11 was investigated using a combination of physiological characterization, genomics, and transcriptomics. Exposure to 100 mg/L of Mg–Fe-LDH NPs led to NP adsorption onto Aodex, increased expression of *Aodex*, and generation of a new Shine-Dalgarno sequence (GGGAG) and sRNAs that both influenced the expression of *Aodex*. Moreover, the expressions of transcripts related to ferric iron metabolic functions were significantly influenced by treatment.

**Conclusions:**

These results provide valuable information for further investigation of the *A. oxidans* KQ11 response to Mg–Fe-LDH NPs and will aid in achieving improved marine dextranase production, and even improve such activities in other marine microorganisms.

**Electronic supplementary material:**

The online version of this article (10.1186/s13068-019-1528-2) contains supplementary material, which is available to authorized users.

## Background

Nanotechnology is a transformative tool that can be used to develop and enhance high-value products from renewable and biocompatible raw materials [1]. Nanoparticles (NPs) have attracted considerable interest due to their unique optical, electronic, and magnetic characteristics relative to their bulk counterparts [2]. Moreover, NPs are widely used in commercial products owing to their versatile properties, including surface areas, particle sizes, and quantum effects [3]. Layered double hydroxide (LDH) NPs, also known as anionic clay or hydrotalcite, are a family of inorganic lamellar materials with positively charged brucite-like layers comprising mixed metal hydroxides that are defined by the general formula [M_1− *X*_^II^M_*X*_^III^(OH)_2_]^*X*+^(A^*n*−^)_*X*/*n*_·mH_2_O. Here, M^II^ is a divalent cation, M^III^ is a trivalent metal cation, *x* is the molar ratio of the trivalent cation [M^III^/(M^III^ + M^II^)], and A^*n*−^ is a gallery anion with charge *n* [4–6]. LDHs have received widespread attention in diverse applications, including in catalysis [7], polymer nanocomposites [8], pharmaceuticals [9, 10], and sensors [11]. Concomitantly, environmental pollution has emerged as an important problem over the last couple of decades, and interest is growing in using LDHs to remove environmental contaminants (e.g., heavy metals, pesticides, and polycyclic aromatic hydrocarbons) [12–15]. The large surface areas of LDHs play a vital role in enhancing the kinetics of electrochemical reactions and providing a large number of active sites for desired electrochemical reactivities [16].

In addition to the above, the investigation of LDH NP interactions with bacteria is of increasing interest. Numerous studies have shown that NPs can improve antimicrobial, anticorrosion, and antitumor functionalities through silver NPs [17], copper NPs [1, 18], and LDH NPs [19]. Moreover, other studies have shown that NPs can improve the growth of bacterial cells and their production of metabolites [20–22]. Specifically, NP aggregates can attach to and/or entrap cells, thereby impacting their cellular functions. It should also be noted that variation exists in the metabolite production by different microorganisms, including *Escherichia coli*, *Bacillus*, *Bacillus subtilis,* and *Nocardiopsis* sp., as indicated by their different production capacities and qualities [20, 23]. Al-Zn-LDH and Mg–Al-LDH [18, 24] LDHs have been intensively studied recently, while Mg–Fe-LDH has been much less investigated [6]. However, Mg–Fe-LDH NPs, which have been trademarked as Alpharen and Fermagate, have been intensively investigated in animal and clinical trials in the treatment of hyperphosphatemia in hemodialysis patients. Such studies have provided strong evidence of their high phosphate removal efficiency and biocompatibility [25, 26]. Moreover, increasing evidence indicates that Al can exert neurological, skeletal, and hematological toxicity. Consequently, the development of Al-free LDHs capable of maintaining highly efficient gene delivery has become increasingly desirable [26].

Few investigations have been conducted to evaluate the influence of LDHs on marine microorganisms. Indeed, investigation of LDH toxicity to microorganisms has been almost entirely conducted on terrestrial microorganisms, including *Pseudomonas aeruginosa*, *Staphylococcus aureus*, *B. subtilis*, and others [27–29]. However, most of the toxicities involved cell damage after analysis, which is likely resultant from low salinity tolerance of terrestrial microorganisms relative to marine microorganisms. It is well documented that marine microorganisms have high salt tolerance, hyperthermostability, barophilicity, alkali resistance, and low optimal growth temperatures. In addition, the intercalated molybdate anion can slowly diffuse out of the inner structure of LDHs in a controlled manner, resulting in relatively long-lived corrosion inhibition effects in marine anticorrosion applications [18].

Dextranases have drawn considerable attention due to their high potential for application in various fields, including in medical, dental, and sugar industries [30–36]. Dextranases (α-1,6-D-glucan 6-glucanohydrolase; EC 3.2.1.11) hydrolyze dextran to oligosaccharides at the α-1,6 glucosidic bond and are members of the glycoside hydrolase families (GH) 49 and 66 based on amino acid sequence homology [32, 37–39]. *A. oxidans* KQ11 was previously isolated by our research group from the Yellow Sea in the Lianyungang coastal region of China and produces dextranase (Aodex, Protein Data Bank code 6NZS) [36]. Here, we present a systematic study of the effects of repetitive dosing of various concentrations of Mg–Fe-LDH NPs on the marine bacterium *A. oxidans* KQ11 and its ability to produce Aodex. Transcriptional regulation is the mechanistic basis for bacterial growth and metabolism, and genome-wide transcriptional profiling can improve our understanding of the mechanisms underlying physiological changes [40, 41]. Consequently, transcriptional and genomic profiling was used to evaluate the mechanisms underlying variation in Aodex production following Mg–Fe-LDH NP exposure.

## Results

### Bacterial growth and Aodex production by *A. oxidans* in the presence of Mg–Fe-LDH NPs

**Fig. 1:**
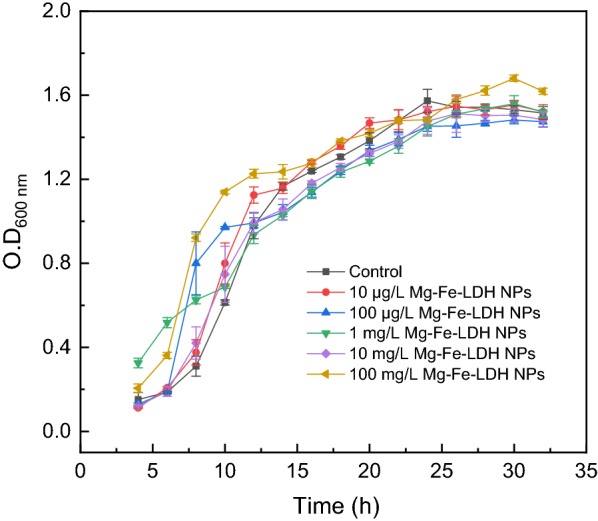
Growth curves of *A. oxidans* KQ11 batch cultures that were chronically exposed to different concentrations of Mg–Fe-LDH NPs

To determine the influence of Mg–Fe-LDH NPs on *A. oxidans* KQ11 growth and Aodex production, these properties were analyzed in the presence of varying concentrations of Mg–Fe-LDH NPs. When Mg–Fe-LDH NPs were added to bacterial cultures, statistically different levels of bacterial growth were not observed after 32 h (Fig. 1). This result was evident even at higher Mg–Fe-LDH NP concentrations (100 mg/L), implying that Mg–Fe-LDH NPs (at concentrations ≤ 100 mg/L) did not result in obvious growth effects on *A. oxidans* KQ11.

**Fig. 2:**
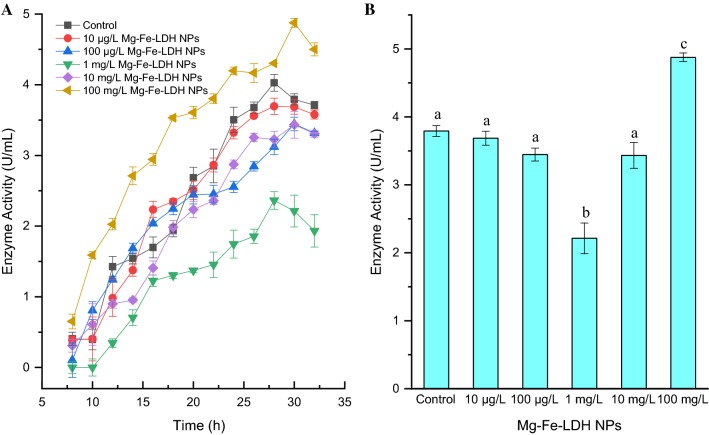
Aodex generation after exposure of *A. oxidans* KQ11 to different concentrations of Mg–Fe-LDH NPs (**A**) and at 30 h (**B**). Columns with different letters indicate statistically significant differences (*p* < 0.05) between controls and treatments. The error bars represent standard deviations of averages (n = 3)

In contrast, enzyme production by *A. oxidans* KQ11 changed as a result of exposure to Mg–Fe-LDH NPs (Fig. 2A). Specifically, when exposed to 10 μg/L Mg–Fe-LDH NPs over 32 h, Aodex production was similar to that of the control and peak enzyme production occurred at 28 h, which was consistent with previous results [37]. The activities of Aodex upon exposure to 10 μg/L Mg–Fe-LDH NPs and the control were 3.69 U/mL and 4.03 U/mL, respectively. Exposure to increasing Mg–Fe-LDH NPs concentrations in the range of 10 μg/L to 1 mg/L resulted in decreased enzyme production, with Aodex production exhibiting the lowest activity after exposure to 1 mg/L Mg–Fe-LDH NPs. Peak enzyme production after exposure to Mg–Fe-LDH NPs at concentrations of 10 μg/L, 100 μg/L, and 1 mg/L was 3.69 U/mL at 28 h, 3.45 U/mL at 30 h, and 2.36 U/mL at 28 h, respectively. As increasing exposure to Mg–Fe-LDH NPs concentrations occurred beyond 1 mg/L, the overall trend of enzyme production began to rapidly increase. Indeed, enzyme production after exposure to 10 mg/L Mg–Fe-LDH NPs was similar to that after exposure to 100 μg/L Mg–Fe-LDH NPs, with a peak enzyme production of 3.43 U/mL at 30 h. Moreover, enzyme production was highest over the examined concentration range (10 μg/L to 100 mg/L) when exposed to an Mg–Fe-LDH NP concentration of 100 mg/L. Specifically, enzyme production in this treatment was 4.88 U/mL at 30 h, which was about 21.1% higher than the control, although peak enzyme production was delayed 2 h. Enzyme production after exposure to different Mg–Fe-LDH NP concentrations for 30 h was separately analyzed in order to directly compare enzyme production results (Fig. 2B), which also confirmed the above results. Briefly, enzyme production after exposure to Mg–Fe-LDH NPs concentrations in the range of 10 μg/L to 1 mg/L gradually declined, while enzyme production after exposure to Mg–Fe-LDH NPs concentrations in the range of 1 mg/L to 100 mg/L rapidly rebounded. The U-shaped trend of Aodex production resulted from the competition between Mg–Fe-LDH NP adsorption and the promotion of Aodex expression after exposure to NP treatment concentrations of 10 μg/L–100 mg/L.

### Characterization of bacterial cell morphology after exposure to Mg–Fe-LDH NPs

**Fig. 3:**
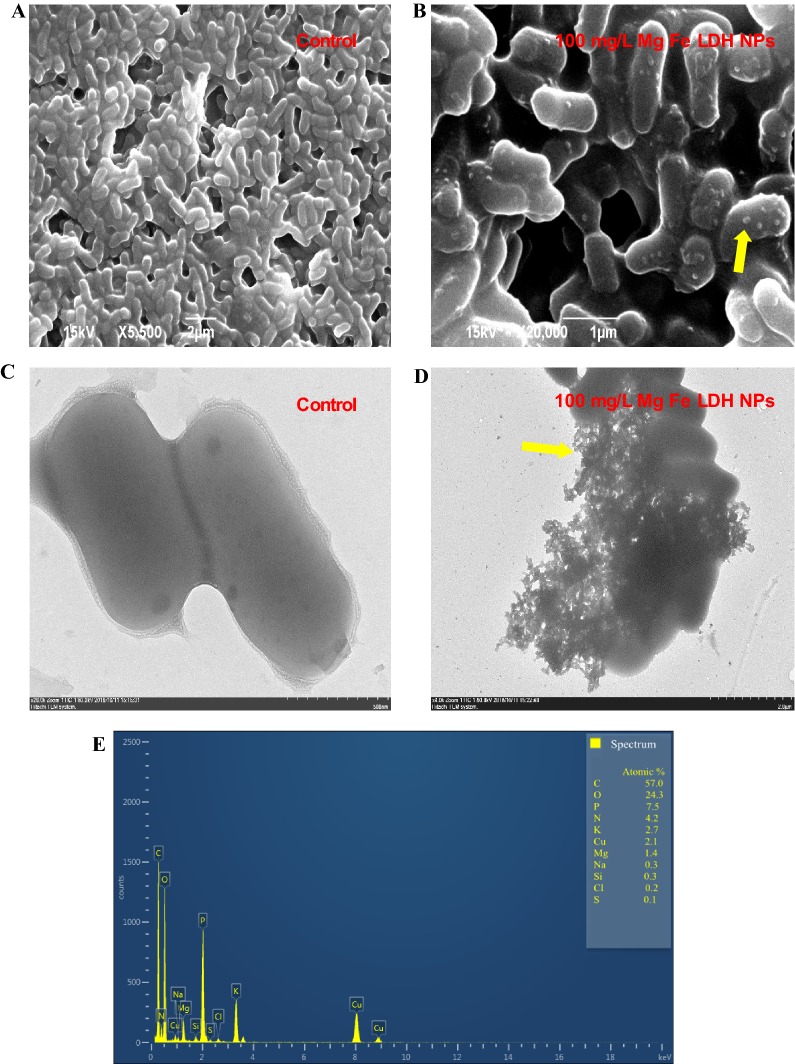

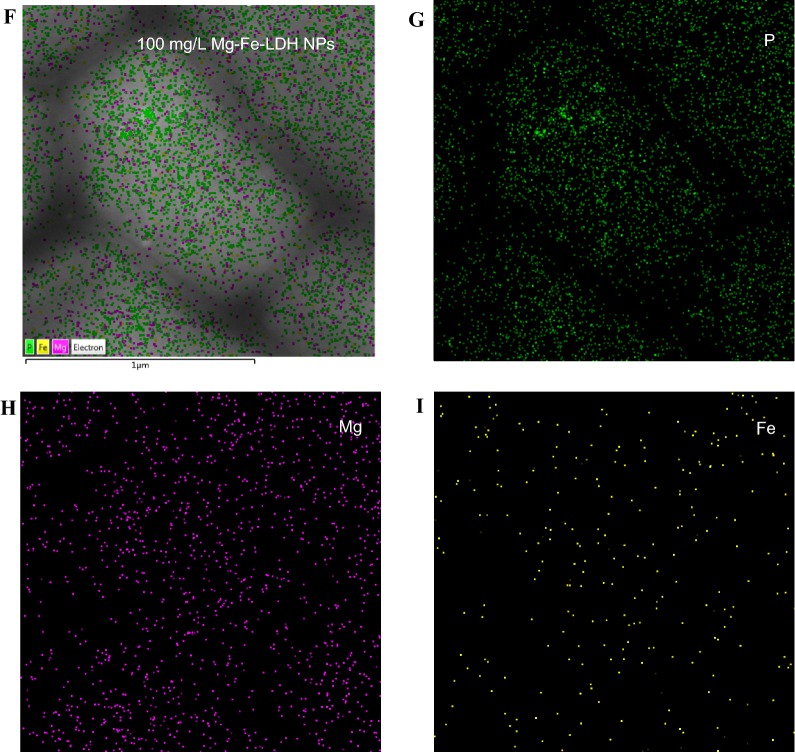
Characterization of *A. oxidans* KQ11 cell structure after exposure to 100 mg/L of Mg–Fe-LDH NPs for 30 h using SEM (**A**, **B**), TEM (**C**, **D**), EDS (**E**), and EDS mapping (**D**–**I**). The yellow arrows show Mg–Fe-LDH NPs attached to cell surfaces (for interpretation of the color references to color in this figure legend, the reader is referred to the web version of this article)

To gain further insight into the possible effects of Mg–Fe-LDH NPs on *A. oxidans* KQ11 cells, SEM, TEM, EDS, and EDS microscopy were used to examine *A. oxidans* KQ11 cells after exposure to 100 mg/L of Mg–Fe-LDH NPs. SEM (Fig. 3A, B) and TEM (Fig. 3C, D) images revealed that Mg–Fe-LDH NPs adhered to cellular membranes of *A. oxidans* KQ11 after washing cells three times, as noted by visible structures on the surface of *A. oxidans* KQ11 cells. However, alterations in *A. oxidans* KQ11 cell walls were not observed after exposure to 100 mg/L Mg–Fe-LDH NPs including a lack of surface disruptions, shrinkages, and irregularities.

To further determine the nature of the substances adsorbed on cell surfaces, the elemental composition of cell surfaces was investigated using EDS (Fig. 3E). These analyses indicated the presence of magnesium, but a lack of an iron signal, which may be due to the magnetic properties of iron and the low concentration used in these experiments that would render it difficult to detect using EDS. Consequently, EDS mapping was used to further detect the elements associated with cellular surfaces and further evaluate the presence of iron in association with cells (Fig. 3F). EDS mapping indicated the presence of phosphorus (Fig. 3G; green), magnesium (Fig. 3H; pink), and iron (Fig. 3I; yellow), thereby confirming that Mg and Fe were adhered to the surface of bacterial cells and implying that the structures on the surface of *A. oxidans* KQ11 were Mg–Fe-LDH NPs. The Aodex production experiments indicated that exposure to 100 mg/L of Mg–Fe-LDH NPs may influence cell membrane permeability, metabolite production, and/or gene expression in *A. oxidans* KQ11 cells, which would interact with cellular components to alter cellular processes. Mg–Fe-LDH NPs can likely penetrate cell membranes and reach cytosolic compartments due to their ability to dissolve slowly while releasing Mg^2+^ and Fe^3+^ ions. Nevertheless, understanding the exact mechanism by which Mg–Fe-LDH NPs improved Aodex production by *A. oxidans* KQ11 required further investigation.

### Characterization of Mg–Fe-LDH NP endocytosis by *A. oxidans* KQ11

**Fig. 4:**
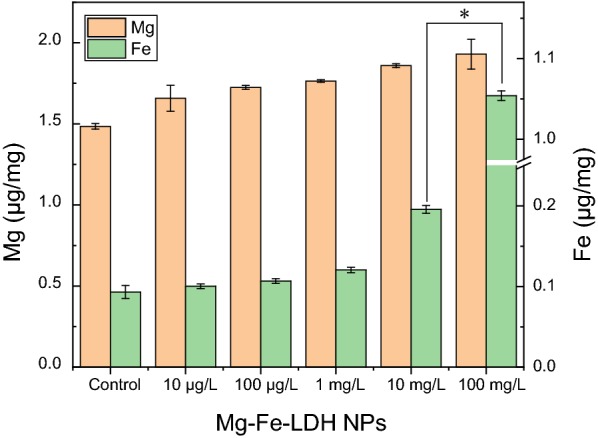
Detection of intracellular Mg and Fe ion concentrations in *A. oxidans* KQ11 using ICP-AES after exposure to different concentrations of Mg–Fe-LDH NPs. Asterisks (*) represent statistically significant (*p *< 0.05) differences

As described above, the primary influence of Mg–Fe-LDH NPs on enzyme production by *A. oxidans* KQ11 cells was through physical interactions with cells, and/or exposure to metal ions released from Mg–Fe-LDH. Thus, ICP-AES was used to measure the total intracellular metal ion content within *A. oxidans* KQ11 cells in order to identify the contribution of metal ions to the physiological influence of Mg–Fe-LDH NPs. The relative distribution and concentration of heavy metals within *A. oxidans* KQ11 cells of the Mg–Fe-LDH NP-treated bacterial cultures are shown in Fig. 4. Mg and Fe concentrations were all higher within *A. oxidans* KQ11 cells when exposed to Mg–Fe-LDH NP concentrations in the range of 10 μg/L to 100 mg/L. Furthermore, the concentration of Fe was significantly higher within *A. oxidans* KQ11 cells when exposed to 100 mg/L of Mg–Fe-LDH NPs than in cells of other treatment groups or the control group.

### Transcriptional profiling of the *A. oxidans* KQ11 response to Mg–Fe-LDH NP exposure

The transcriptional response of *A. oxidans* KQ11 cells in response to Mg–Fe-LDH NPs was investigated with RNA-seq sequencing on the Illumina HiSeq platform. A total of 16.2 GBp of clean sequence read data were generated from the control and treatment samples. Specifically, gene-mapped transcript reads were generated three control (Control1, Control2, and Control3) and three Mg–Fe-LDH treatment (Mg–Fe-LDH1, Mg–Fe-LDH2, and Mg–Fe-LDH3) libraries, respectively (Additional file 1: Table S2). All unigenes were annotated using several databases including the CAZy, COG, GO, KEGG, NR, PFAM, and SwissProt databases (Additional file 1: Table S3). Non-redundant genes were obtained from all experimental samples and used as a transcriptome database to identify differentially expressed genes (DEGs, fold change > 2, *p* < 0.05) between controls samples and Mg–Fe-LDH-treated samples. These analyses indicated that 23 DEGs were up-regulated and 47 were down-regulated due to Mg–Fe-LDH exposure (Additional file 1: Table S4).

## Discussion

Dextranases hydrolyze dextran to oligosaccharides at the α-1,6 glucosidic bond resulting in the production of isomaltose, isomaltotriose, small amounts of d-glucose, and traces of large oligomers as the primary products of the hydrolysis reaction. Previously, we isolated a dextranase from the marine bacterium *A. oxidans* KQ11 (Aodex; NCBI-n: JX481352.1) that was collected from Yellow Sea sediments near Lianyungang, China [37]. Aodex is active at low temperatures, is rapidly produced, and is stabile under alkaline conditions [37]. In addition, this enzyme derived from marine organisms has a characteristically high salinity tolerance and a low ideal temperature, conferring better application potential than homologous enzymes from terrestrial counterparts.

In this study, the growth of *A. oxidans* KQ11 after exposure to different concentrations of Mg–Fe-LDH NPs was not significantly influenced, while Aodex production was completely different among treatments. Specifically, enzyme production after exposure to Mg–Fe-LDH NPs at concentrations in the range of 10 μg/L to 1 mg/L gradually decreased with increasing concentration exposures. However, enzyme productions after exposure to Mg–Fe-LDH NPs concentrations in the range of 1 mg/L to 100 mg/L rapidly rebounded. To clarify the mechanism of Mg–Fe-LDH NP influence on *A. oxidans* KQ11 physiology, morphological and ultrastructural changes of cells were examined. These results indicated that Mg–Fe-LDH NPs could adhere to *A. oxidans* KQ11 cell membranes, but alterations in *A. oxidans* KQ11 cell walls were not observed, including a lack of surface disruptions, shrinkages, and irregularities after exposure to Mg–Fe-LDH NPs. Most marine microorganisms have unique physiological properties due to their unique environments compared to their terrestrial counterparts including high salt tolerance, hyperthermostability, barophilicity, alkali-resistance, and low optimum growth temperatures. Such characteristics could explain why *A. oxidans* KQ11 cell damage was not observed after exposures to such high concentration of Mg–Fe-LDH NPs. Concomitant to Aodex production rapidly increasing after exposure to 100 mg/L Mg–Fe-LDH, intracellular Fe concentrations were significantly higher in *A. oxidans* KQ11 after exposure to 100 mg/L Mg–Fe-LDH NPs when compared to the other treatment and control groups.

We previously described the structure of Mg–Fe-LDH NPs and their adsorption onto dextranase [6, 26]. Considering the gradual decrease in Aodex enzyme production after exposure to Mg–Fe-LDH NPs concentrations in the range of 10 μg/L to 1 mg/L, XRD (Additional file 1: Figure S1A) and FTIR (Additional file 1: Figure S1B) were used to investigate the adsorption onto Aodex by Mg–Fe-LDH NPs. The XRD spectra pattern of the Aodex/Mg–Fe-LDH biohybrid displayed characteristic diffraction peaks, with peak broadening and decreased intensity indicative of reduced crystallinity characteristics of the biohybrid. The FTIR spectra of the Mg–Fe-LDH NPs, Aodex, and Aodex/Mg–Fe-LDH NPs are shown in Additional file 1: Figure S1. The spectra exhibited broad and intense bands between 3900 and 2700/cm that were associated with the stretching of hydrogen-bonded hydroxyl groups from both the hydroxide layers and interlayered water molecules [24]. The shoulder located at about 3000/cm can be attributed to hydrogen bonding between water and anions located in the interlayer spacing including C–H, C–O–C, and C–O stretching bands [24, 42]. The bands at about 1650 and 1550/cm were assigned to the amide groups of amino acids, including C=O and N–H stretching bands. These results confirmed that Aodex was adsorbed on the surface of the Mg–Fe-LDH NPs, while also suggesting that the protein retained its secondary structure and did not denature. Thus, Aodex can adsorb on the surface of Mg–Fe-LDH NPs. Such characteristics provide a foundation for further utilization of Aodex, and especially in sugar industry applications, owing to the ease of release and effective removal of dextran generated during the production process. Previous studies have reported that LDHs can effectively immobilize numerous biomolecules or enzymes on their structures including laccase [43], polyphenol oxidase [44], urease [45], acid phosphatase–polyphenol oxidase [46], cytochrome c nitrite reductase [47], and horseradish peroxidase [48]. These characteristics enable their application in environmental pollutant monitoring including of cyanide, phenol derivatives, urea, azides, As (V), fluoride, nitrite, and H_2_O_2_.

**Fig. 5:**
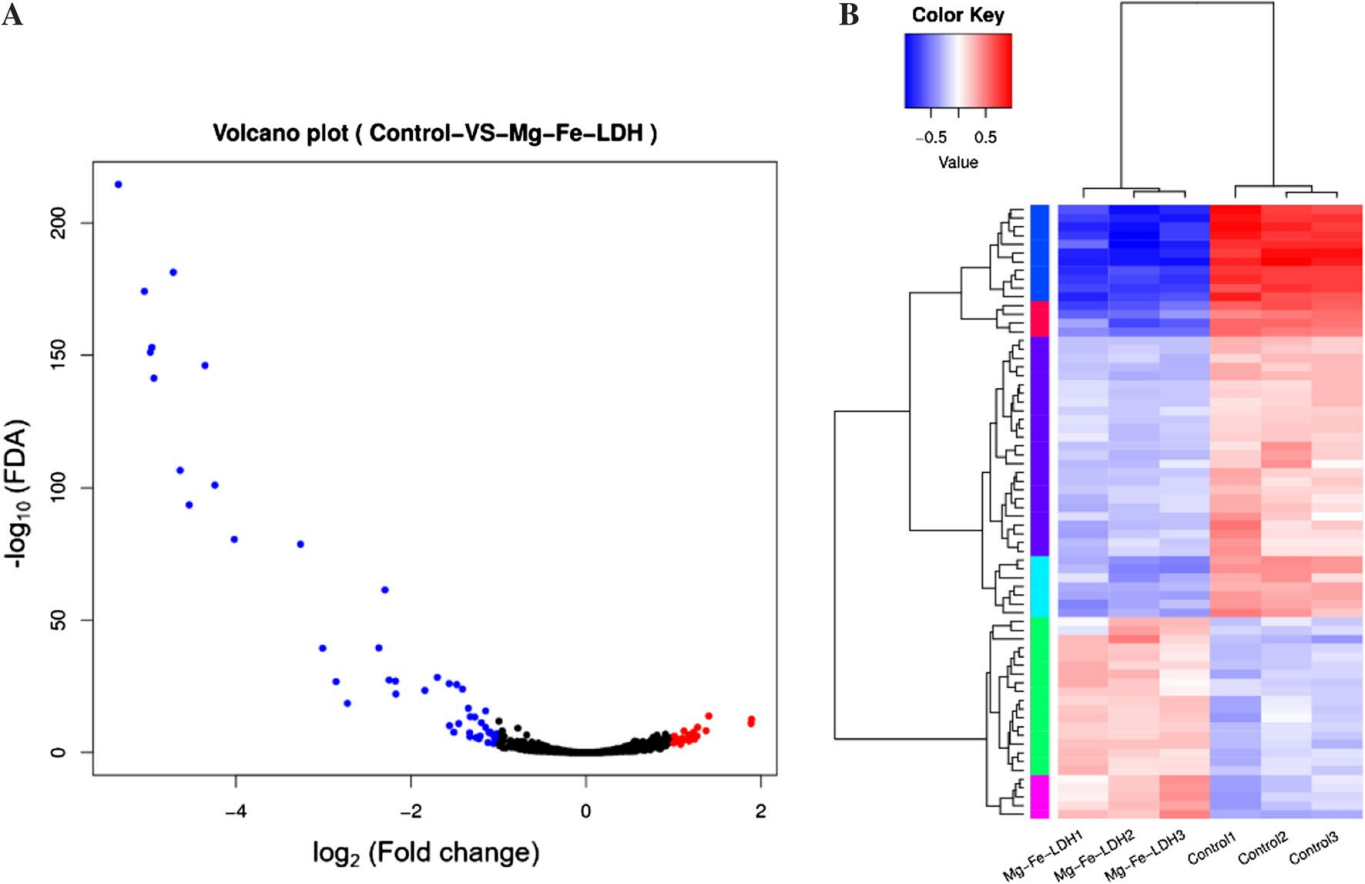
Changes in the gene expression of *A. oxidans* KQ11 cells induced by Mg–Fe-LDH treatment. **A** Significantly up-regulated genes are shown in red, down-regulated genes in blue, and those not exhibiting significant differences in expression are shown as black dots. The abscissa represents the fold changes in gene expression among different samples, and the ordinate represents the statistical significance of differences in expression change. **B** Heat map and clustering analysis of transcriptional profiles of genes encoding transcription factors. High expression levels are depicted in red, and low expression levels in blue. Clustering was conducted using log10 (FPKM + 1) values after normalization of expression values

While Mg–Fe-LDH NPs adsorbed the Aodex, Mg–Fe-LDH NPs also released Fe ions that could regulate Aodex production of *A. oxidans* KQ11. Thus, Mg–Fe-LDH NPs could contribute to more straightforward Aodex production via the above mechanism. Furthermore, this interaction could explain why Aodex production rapidly rebounded after exposure to 100 mg/L. To investigate the molecular mechanism underlying the regulation of Aodex productions by Mg–Fe-LDH, transcriptional profiling was conducted. A total of 4563 genes were expressed, and the expected number of Fragments Per Kilobase of transcript sequence per Millions base pairs sequenced (FPKM) was used to identify genes that were differentially expressed. Using the criteria of a twofold change in expression and an FDR *p* value < 0.005, a total of 70 differentially expressed genes were identified. Of these, 23 genes were up-regulated and 47 were down-regulated in *A. oxidans* KQ11 after exposure to Mg–Fe-LDH NPs. The most important genes among those that were down-regulated included those that encoded siderophore synthetase components, iron complex transport system permease proteins, iron complex transport system ATP-binding proteins, NADPH-dependent ferric siderophore reductases, Fe-S cluster assembly ATP-binding proteins, and the Fe-S cluster assembly protein SufD, which are all directly related to inorganic ion transport and metabolism. The most important genes among those that were up-regulated included those that encoded N-acetylglucosamine-6-phosphate deacetylases, phosphotransferase system IIC components, ATPase components, predicted arabinose efflux permeases, sarcosine oxidase gamma subunits, 2,4-dienoyl-CoA reductases, formyltetrahydrofolate hydrolases, phosphotransferase system IIB components, 6-phosphogluconolactonase/glucosamine-6-phosphate isomerase/deaminases, sugar lactone lactonases, and malate synthases, which are all directly related to materials (carbohydrates, amino acids, and nucleotide) transport, metabolism, energy production and conversion, and signal transduction. The overall pattern of differential expression of transcription factor genes is shown in Fig. 5.

**Fig. 6:**
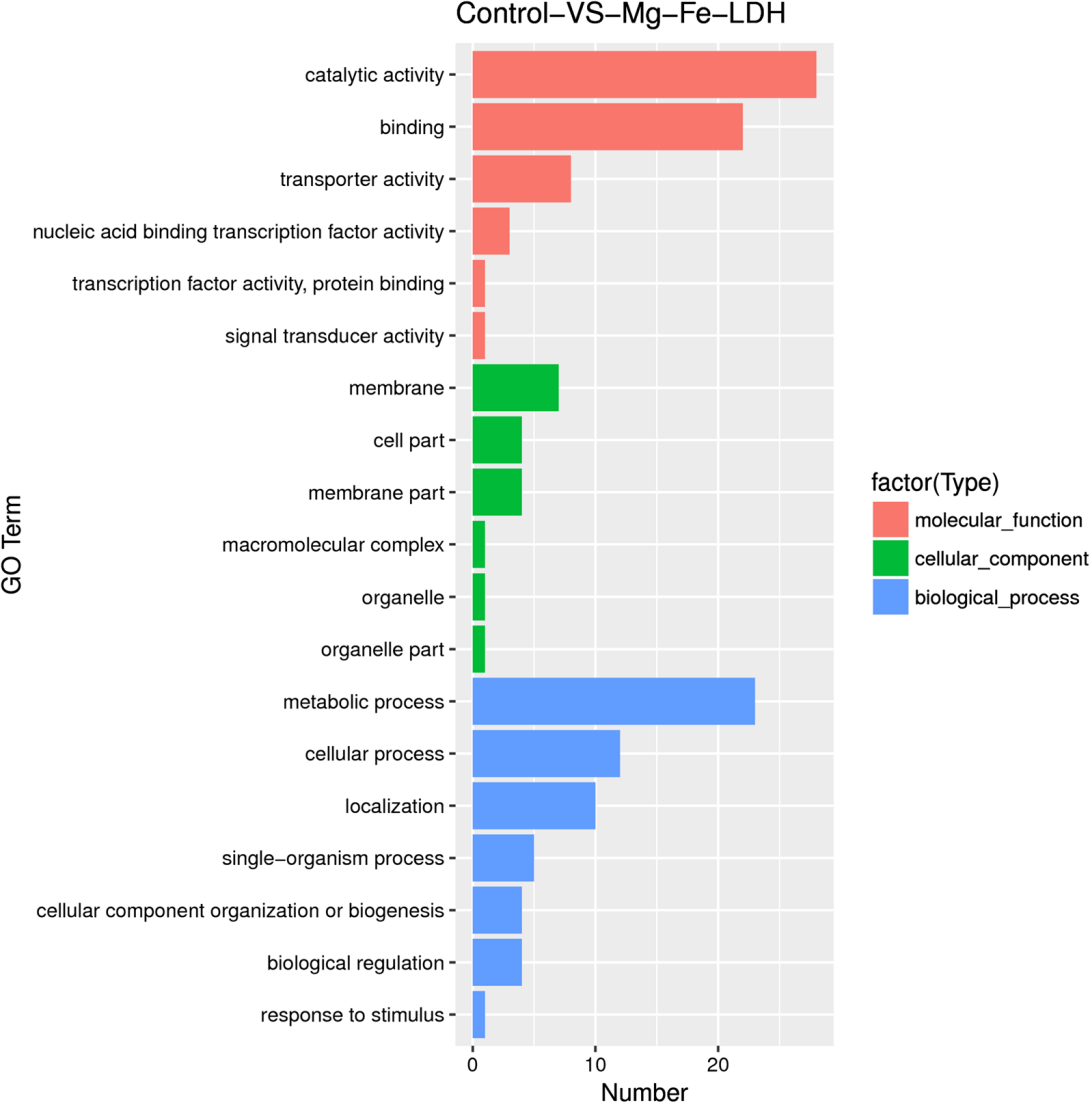
GO enrichment analysis of differentially expressed genes of *A. oxidans* KQ11 after Mg–Fe-LDH treatment

GO analysis was also performed on the DEGs (Fig. 6). The DEGs all belonged to several categories, including cellular components, molecular functions, and biological processes. The majority of DEGs classified into the molecular function category were represented by those involved in catalysis, binding, and transportation activities. Among the DEGs classified into the cellular components category, membranes, cell parts, and membrane parts were prominently represented. Of the DEGs classified within the biological processes category, the vast majority were related to metabolic processes, cellular processes, localization, single-organism processes, cellular component organization or biogenesis, and biological regulation. The number of DEGs categorized as being involved in cellular components was lower than that of DEGs involved in molecular functions and biological processes (Fig. 6), which was consistent with the lack of observed cell damage in the experiments. Notably, 195 DEGs were classified as being involved in catalytic activity, while 70 were annotated as being involved in response to exposure, and 13 were annotated as being involved in ferric iron metabolism functions (particularly Fe^3+^ transport systems). These results provide a valuable framework for future studies of the response of *A. oxidans* KQ11 to Mg–Fe-LDH NP exposure.

**Fig. 7:**
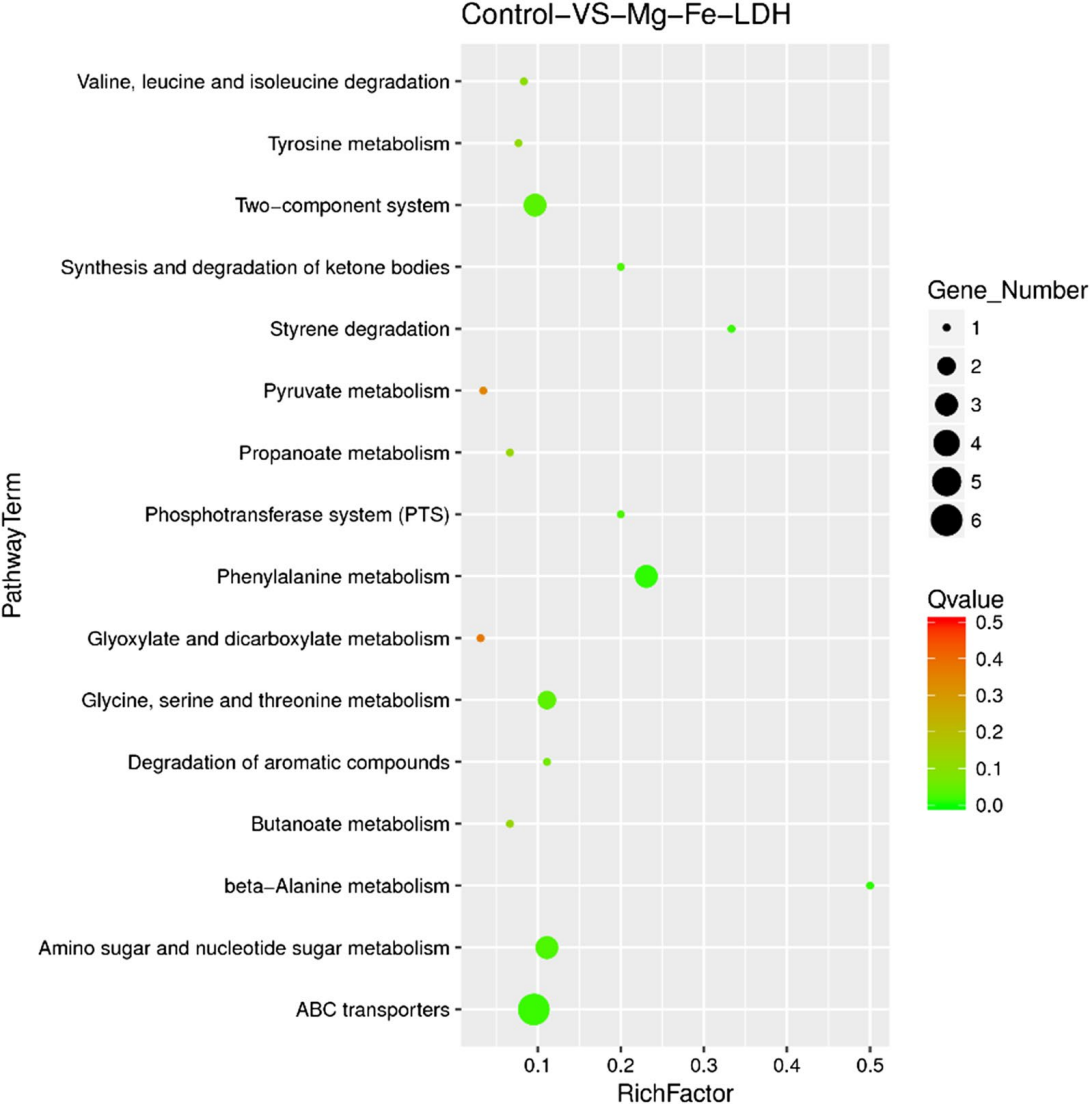
KEGG pathways represented by enriched differentially expressed genes

The metabolic pathways coinciding with the DEGs were identified and analyzed using the KEGG database. The 16 most enriched pathways are shown in Fig. 7 and Table 1. Among the identified metabolic pathways, DEGs were primarily involved in the metabolism of biomolecules including carbohydrates and amino acids. Six DEGs were associated with ABC transporter pathways. Interestingly, DEGs involved in several carbohydrate transport and metabolism pathways including those associated with amino sugar and nucleotide sugar metabolism (3), phosphotransferase system (1), and glyoxylate and dicarboxylate metabolism (1) pathways were up-regulated in *A. oxidans* KQ11 after Mg–Fe-LDH NP treatment.

**Table 1:** Overview of DEGs involved in KEGG pathway

No.	Pathway ID	DEGs with pathway annotation (19)	All genes with pathway annotation (577)	KEGG pathway	*p* value	*Q* value	Gene list	KO list
1	ko02020	3 (15.79%)	31 (5.37%)	Two-component system	0.015012085	0.03940672	KQ11_GM000490, KQ11_GM000492, KQ11_GM000491,	K07793, K07795, K07794
2	ko00360	3 (15.79%)	13 (2.25%)	Phenylalanine metabolism	0.000500975	0.01052047	KQ11_GM003846, KQ11_GM003870, KQ11_GM001828,	K00276, K00146, K05710
3	ko00260	2 (10.53%)	18 (3.12%)	Glycine, serine and threonine metabolism	0.018086604	0.04220208	KQ11_GM003846, KQ11_GM000418,	K00276, K13745
4	ko01220	1 (5.26%)	9 (1.56%)	Degradation of aromatic compounds	0.032243512	0.06771138	KQ11_GM001828,	K05710
5	ko02060	1 (5.26%)	5 (0.87%)	Phosphotransferase system (PTS)	0.009694457	0.02908337	KQ11_GM003720,	K02804
6	ko02010	6 (31.58%)	63 (10.92%)	ABC transporters	0.002328422	0.01588777	KQ11_GM003996, KQ11_GM003998, KQ11_GM003995, KQ11_GM000625, KQ11_GM000624, KQ11_GM000623,	K02015, K02016, K02013, K02012, K02011, K02010
7	ko00630	1 (5.26%)	32 (5.55%)	Glyoxylate and dicarboxylate metabolism	0.284432368	0.37331748	KQ11_GM000606,	K01638
8	ko00620	1 (5.26%)	29 (5.03%)	Pyruvate metabolism	0.246513313	0.34511864	KQ11_GM000606,	K01638
9	ko00643	1 (5.26%)	3 (0.52%)	Styrene degradation	0.003026241	0.01588777	KQ11_GM003870,	K00146
10	ko00520	3 (15.79%)	27 (4.68%)	Amino sugar and nucleotide sugar metabolism	0.009121947	0.02908337	KQ11_GM003720, KQ11_GM003723, KQ11_GM003721,	K02804, K01443, K02564
11	ko00650	1 (5.26%)	15 (2.60%)	Butanoate metabolism	0.083581921	0.12537288	KQ11_GM000543,	K01029
12	ko00410	1 (5.26%)	2 (0.35%)	Beta-Alanine metabolism	0.00102903	0.01080481	KQ11_GM003846,	K00276
13	ko00280	1 (5.26%)	12 (2.08%)	Valine, leucine and isoleucine degradation	0.055721077	0.1063766	KQ11_GM000543,	K01029
14	ko00640	1 (5.26%)	15 (2.60%)	Propanoate metabolism	0.083581921	0.12537288	KQ11_GM000991,	K18382
15	ko00350	1 (5.26%)	13 (2.25%)	Tyrosine metabolism	0.064571367	0.11299989	KQ11_GM003846,	K00276
16	ko00072	1 (5.26%)	5 (0.87%)	Synthesis and degradation of ketone bodies	0.009694457	0.02908337	KQ11_GM000543,	K01029

The Shine-Dalgarno sequence is a ribosomal binding site in bacterial and archaeal mRNA that is generally located around eight bases upstream of the start codon, AUG [49]. The sequence is also present in some chloroplast and mitochondrial transcripts. The RNA sequence helps recruit ribosome to mRNA in order to initiate protein synthesis and align the ribosome with the start codon. Once recruited, tRNA molecules can add amino acids sequentially, as dictated by the codons and moving downstream from the translational start site. The six-base consensus sequence is AGGAGG and AGGAGGU in *E. coli*, for example, although the subsequence GAGG dominates in *E. coli* virus T4 early genes [49]. The Shine-Dalgarno sequences of *A. oxidans* KQ11 RNA were predicted with RBSfinder after treatment with Mg–Fe-LDH NPs (Additional file 1: Table S5). A key difference was observed in the Shine-Dalgarno sequences with and without treatment, wherein the Shine-Dalgarno sequence of the Aodex (GeneID: KQ11_GM001677) position was 1,772,726 with the original start codon of CTA, but at a new starting position of 1,772,624 after treatment. Thus, the original sequence start coordinate moved upstream within the same reading frame. The new stop position also moved to 1,774,612 after treatment of *A. oxidans* KQ11 with Mg–Fe-LDH NPs and the pattern of the Shine-Dalgarno sequence changed to GGGAG, with the start codon changing from CTA to ATG. The transcriptional profile of *A. oxidans* KQ11 in the absence (control) and presence of Mg–Fe-LDH indicated that Aodex was up-regulated with an FDR *p* value of ~ 0.057. Thus, the GGGAG pattern of the Shine-Dalgarno sequence likely plays an important role in Aodex transcription and expression. Several studies have shown that base pairing between the Shine-Dalgarno sequence in mRNA and the 3′ end of 16S rRNA is critical for initiation of translation by bacterial ribosomes [50, 51]. Thus, mutations in Shine-Dalgarno sequence can reduce or increase translational responses in prokaryotes [52]. These changes are due to reduced or increased mRNA-ribosome pairing efficiencies, as evidenced by the restoration of translation by compensatory mutations in the 3′-terminal of 16S rRNA sequences.

**Fig. 8:**
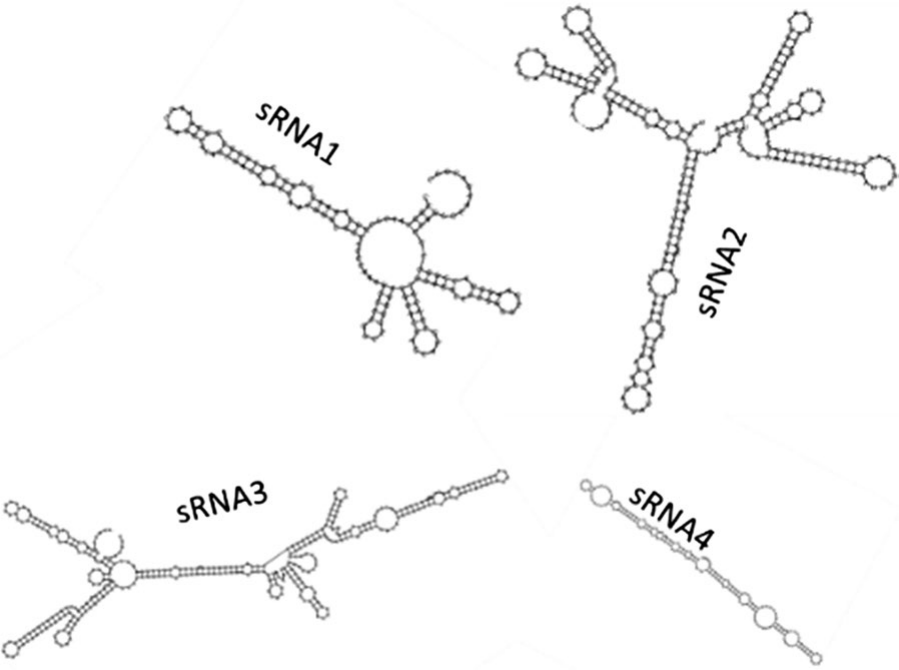
Secondary structures of sRNA1, sRNA2, sRNA3, and sRNA4 of *A. oxidans* KQ11 following Mg–Fe-LDH NP treatment. The sRNAs were located on the plas2, plas3, plas1, and plas1 regions of the *A. oxidans* KQ11 chromosome, respectively

**Fig. 9:**
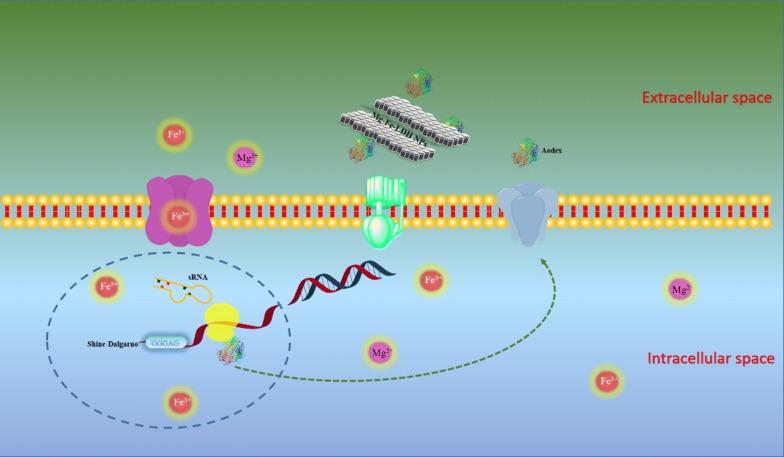
Proposed mechanism of the effects of Mg–Fe layered double hydroxide nanoparticles on the physiological functioning of *A. oxidans* KQ11

Bacterial small RNAs (sRNA) are 50- to 500-nucleotide-long non-coding RNA molecules produced by bacteria that are highly structured and contain several stem loops [53]. Bacterial sRNAs affect how genes are expressed within bacterial cells via interaction with mRNAs or proteins and thus can affect a variety of bacterial functions like metabolism, virulence, environmental stress responses, and cell structures [54, 55]. Consequently, bacterial sRNAs exhibit a wide range of regulatory mechanisms. Generally, sRNAs bind to protein targets and modify the functions of bound proteins [56]. Alternatively, sRNAs can interact with mRNA targets and regulate gene expression by binding to complementary mRNAs and blocking translation, or by unmasking or blocking ribosome-binding sites. Many sRNAs are involved in the regulation of stress response [57] and are expressed under stress conditions such as cold shock, iron depletion, the onset of the SOS response, and sugar stress [58]. For example, the small RNA nitrogen stress-induced RNA 1 (NsiR1) is produced by cyanobacteria under nitrogen deprivation conditions [59]. In addition, cyanobacterial NisR8 and NsiR9 sRNAs could be involved in the differentiation of nitrogen-fixing cells (heterocysts) [60]. Novel non-coding sRNA transcripts in *A. oxidans* KQ11 intergenic regions that were expressed after Mg–Fe-LDH NP treatment were annotated by NR. A total of four sRNAs were identified, and their secondary structures were further predicted (Additional file 1: Table S6 and Fig. 8). These sRNAs may affect the expression of *A. oxidans* KQ11 intracellular proteins, and particularly the production of Aodex. Moreover, we hypothesize that these sRNAs are involved in the function of the riboswitches or the efficiency of Aodex production, which was up-regulated after Mg–Fe-LDH NP treatment (Fig. 9).

## Conclusions

Our results indicated that the mechanism underlying the effects of Mg–Fe-LDH NPs on the physiology of the marine bacterium *A. oxidans* KQ11 could be related to the interaction of Fe^3+^, Shine-Dalgarno GGGAG sequences, and sRNAs (Fig. 8). As shown in Fig. 9, the proposed mechanism first involves adsorption of Mg–Fe-LDH NPs onto the surface of *A. oxidans* KQ11 cells, followed by release of Fe^3+^, which would impact carbohydrate transport and associated carbohydrate metabolisms. Secondly, the Shine-Dalgarno sequence is modified to GGGAG, which could play an important role in Aodex transcription and expression. Alternatively, the generation of sRNAs could interact with Aodex and further promote the expression of Aodex. This process would lead to constant Aodex expression with increasing concentrations of Mg–Fe-LDH NPs, while the adsorption of Mg–Fe-LDH NPs to enzymes would concomitantly gradually reach saturation. Such a mechanism would explain the U-shaped trend of Aodex production with increasing concentrations of Mg–Fe-LDH NPs. Nevertheless, abiotic stresses are undoubtedly complex in nature. However, understanding the full potential of biotechnological approaches can provide an important framework for improving enzyme production. Rapidly developing technologies including transcriptome profiling and nanotechnology provide promising future prospects for the development of designed enzymes that exhibit higher efficiency of natural resource utilization and improved productivity under stressful conditions.

The oceans cover more than three quarters of the Earth’s surface and are open ecosystems. The protection of marine environments and the reasonable exploitation and utilization of marine resources are vitally important to the sustainable development of human activities. Recent interest has grown for using LDHs to remove environmental contaminants. In this study, Mg–Fe-LDH NPs enhanced the production of Aodex by a marine bacterium. Furthermore, the mechanism underlying the influence of Mg–Fe-LDH NPs on the marine bacterium *A. oxidans* KQ11 (Fig. 1) was investigated using a combined approach of physiological characterization, genomics, and transcriptomics. These analyses indicated that cellular damage to marine *A. oxidans* KQ11 cells was not observed after Mg–Fe-LDH NP treatment. These results have an important practical significance wherein Mg–Fe-LDH NPs can be applied in the sustainable separation and extraction of marine resources without affecting marine microorganisms, even in marine ecosystems.

## Materials and methods

### *A. oxidans* KQ11 exposure to Mg–Fe-LDH NPs

Freshly grown bacterial colonies on solid nutrient agar medium were inoculated into 50 mL of *A. oxidans* KQ11 culture medium containing 1.0 g/L yeast extract, 5.0 g/L peptone, and NaCl at 4.0 g/L (pH 7.5). Growth was monitored with a UV–visible spectrophotometer at 600 nm until the optical density (OD) reached 0.8. Aliquots (10 µL) of the culture media were then further inoculated in 50 mL of freshly prepared nutrient broth medium containing 1.0 g/L yeast extract, 5.0 g/L peptone, NaCl 4.0 g/L, and 10 g/L dextran 20,000 (dextranase production medium, pH 7.5). The co-precipitation method was used for preparing Mg–Fe-LDH NPs (Additional file 1). In order to ensure Mg–Fe-LDH NPs with given concentrations were completely sterile for the growth and the enzyme production experiments, the Mg–Fe-LDH NP solutions were first sterilized by autoclaving. The Mg–Fe-LDH NPs were then sonicated at 20 kHz in a 100-W bath for 30 min at 25 °C. The Mg–Fe-LDH NP solutions were then sterilized with UV irradiation for 30 min. Subsequently, the Mg–Fe-LDH NPs were sonicated again at 20 kHz in a 100-W bath for 30 min at 25 °C before addition into *A. oxidans* KQ11 culture medium. Aliquots (50 mL) of *A. oxidans* KQ11 culture were exposed to various concentrations of Mg–Fe-LDH NPs (10 μg/L, 100 μg/L, 1 mg/L, 10 mg/L, and 100 mg/L) at the time of inoculation, and growth and enzyme production were monitored at 2-h intervals. All cultures were incubated at 30 °C in an orbital shaker incubator with shaking at 180 rpm and monitoring of bacterial growth in 2-h intervals via OD measurements at 600 nm using a microplate reader (Thermo Scientific™ Multiskan™ FC, Thermo Fisher Scientific Inc., Waltham, MA, USA). Controls consisting of medium without Mg–Fe-LDH NPs were conducted in parallel.

### Dextranase activity assays

Aodex activity was measured using the DNS (3,5-dinitrosalicylic acid) method that is based on the reaction between sugars and the 3,5-dinitrosalicylic acid reagent, as described previously [37, 39]. Briefly, a mixture of 0.05 mL dextranase and 0.15 mL sodium acetate buffer (50 mM) containing 3% dextran 20,000 (pH 5.5) was incubated at 50 °C for 15 min. DNS [61] was added to the experimental and control mixtures to terminate the reactions, and 0.05 mL of enzyme was added to the control group. The mixture was boiled for 5 min, and then, 3 mL of distilled water was added. The absorbance of the mixture was then measured at 540 nm. One unit of dextranase activity was defined as the amount of enzyme that catalyzed the release of 1 μmol of isomaltose (measured as maltose) from dextran 20,000 in 1 min under the specified assay conditions [37]. To establish enzyme production curves, the thallus of the fermentation broth was removed by centrifugation after a specified time of aerobic fermentation and the liquid supernatant was filtered using an ultrafiltrate membrane (crude enzyme). The dextranase activities were then measured at specified times. For adsorbent experiments, the fermentation liquid supernatant recovered after centrifugation was used as the cell-free extract solution. The supernatant containing dextranase was then collected and purified. Briefly, dextranase was purified using a combination of ammonium sulfate fractionation and ion-exchange chromatography. SDS-PAGE and BD-SDS-PAGE (10% w/v SDS-PAGE with 0.5% Blue Dextran) analyses confirmed that the purified Aodex displayed a single band close to the expected molecular weight (66.2 kDa).

### Ultrastructural observations and adsorbent characterization of *A. oxidans* KQ11

The Mg–Fe-LDH NP surface morphologies, physicochemical properties, elemental distribution, and interactions with bacterial cells were all investigated. The Mg–Fe-LDH NP solution was added to the *A. oxidans* KQ11 cultures after sonication (100 W, 20 kHz, 25 °C, 15 min) to obtain bacterial cultures containing Mg–Fe-LDH NPs at concentrations of 10 μg/L, 100 μg/L, 1 mg/L, 10 mg/L, and 100 mg/L. After 30 h of incubation, bacterial cells were collected by centrifugation of cultures for 10 min at 8000×*g* at 4 °C and then washed three times with sterile saline solutions. Scanning electron microscopy (SEM, Hitachi S-4000; Hitachi Instruments Inc., San Jose, CA, USA) and transmission electron microscopy (TEM, Hitachi HT7700; Hitachi Instruments Inc., San Jose, CA, USA) were then conducted on control cells and nanoparticle-treated cultures after suspension overnight in a phosphate-buffered saline (PBS) buffer with 2% glutaraldehyde. The pellet was then washed three times with the PBS buffer. A series of graded ethanol solutions (20%, 50%, 70%, 95%, and 100%) was used for dehydration over three exchanges consisting of 5 min each. STEM, energy-dispersive spectroscopy (EDS) and EDS mapping of *A. oxidans* KQ11 after treatment with 100 mg/L Mg–Fe-LDH NP were conducted using a JEOL 2100F microscope (JEOL, Tokyo, Japan).

Thirty milliliters of the bacterial culture suspensions containing 100 mg/L Mg–Fe-LDH NPs was collected after 30 h of exposure and centrifuged at 8000 rpm for 10 min. The residues were washed a third time with 50 mM PBS (pH 7.0), and cells were washed a third time with ultrapure water and then re-dissolved in 30 mL of ultrapure water. The supernatant following centrifugation was then used to determine the abundance of heavy metals adsorbed onto or into *A. oxidans* KQ11 cells. Heavy metal abundances were determined via inductively coupled plasma atomic emission spectrometry (ICP-AES, iCAP 6300, Thermo Fisher, USA). *A. oxidans* KQ11 suspensions without Mg–Fe-LDH NP exposure were used as controls.

### Genomic and transcriptional analyses [62–67]

The whole genome of *A. oxidans* KQ11 was sequenced prior to transcriptome analyses. The *A. oxidans* KQ11 genome is not discussed in detail here, but annotation of the genome is provided in Additional file 1: Table S1.

#### Prokaryote mRNA sequencing on the Illumina HiSeq platform

RNA-Seq transcriptional profiling of *A. oxidans* KQ11 was conducted for cells in the absence (control) and presence of 100 mg/L Mg–Fe-LDH, with each group including three parallel replicates. Briefly, cells were harvested after exposure to Mg–Fe-LDH for 30 h and then centrifuged at 8000*g* (4 °C) for 10 min. Total cellular RNA of each sample was then extracted using a TRIzol reagent (Invitrogen)/RNeasy Mini Kit (Qiagen). Total RNA was quantified and quality-checked using an Agilent 2100 Bioanalyzer (Agilent Technologies, Palo Alto, CA, USA), NanoDrop spectrophotometer (Thermo Fisher Scientific Inc.), and 1% agarose gel electrophoresis. For subsequent library preparation, 1 μg of total RNA with an RIN value > 7 was used. Next-generation sequencing library preparations were constructed based on the manufacturer’s protocols (NEBNext^®^ Ultra™ Directional RNA Library Prep Kit for Illumina^®^).

Prior to sequencing, rRNA was depleted from total RNA using the Ribo-Zero rRNA Removal Kit (Bacteria) (Illumina). The rRNA-depleted mRNA was then fragmented and reverse-transcribed. First-strand cDNA was synthesized using ProtoScript II Reverse Transcriptase with random primers and actinomycin D. The second-strand cDNA was then synthesized using a second-strand synthesis enzyme mix (including dACGTP/dUTP). The purified double-stranded cDNA was then cleaned using an AxyPrep Mag polymerase chain reaction (PCR) Clean-up kit (Axygen) followed by treatment with an End Prep enzyme mix to repair both ends of the fragments and add dA-tails in a single reaction, followed by T-A ligation to add adaptors to both fragment ends. Size selection of adapter-ligated DNA to recover fragments of ~ 360 bp length (approximate insert size of 300 bp) was then performed using an AxyPrep Mag PCR Clean-up kit (Axygen). The dUTP-containing second strand was then digested with a Uracil-Specific Excision Reagent (USER) enzyme (New England Biolabs). The DNA fragments of each sample were then amplified with PCR over 11 cycles using the P5 and P7 primers, with both primers carrying Illumina-specific sequences that can anneal to the sequencing flow cell and allow bridge PCR, in addition to a P7 primer carrying a six-base index to allow multiplexing. The PCR products were cleaned using an AxyPrep Mag PCR Clean-up kit (Axygen) and quality-checked using an Agilent 2100 Bioanalyzer (Agilent Technologies, Palo Alto, CA, USA) followed by quantification using a Qubit 2.0 Fluorometer (Invitrogen, Carlsbad, CA, USA).

Sequence libraries with different indices were multiplexed and sequenced on an Illumina HiSeq instrument according to manufacturer’s instructions (Illumina, San Diego, CA, USA). Sequencing was conducted with 2 × 150 paired-end (PE) chemistry, while image analysis and base calling were conducted using the HiSeq Control Software (HCS) + OLB + GAPipeline-1.6 (Illumina) on the HiSeq instrument.

#### Data analysis

##### Quality control

Filtering of poor-quality sequence reads including adapters, PCR primers, or fragments thereof, in addition to those with base quality scores < 20 were removed using Cutadapt (v1.9.1).

##### Mapping

Reference genome sequences and gene model annotation files were from genomes of close relatives of *A. oxidans* KQ11. Bowtie2 (v2.1.0) was then used to index the reference genome sequence. Clean sequence data were aligned and mapped to the reference genome using Bowtie2 (v2.1.0).

##### Expression analysis

Transcript sequence data in the FASTA format were first converted from gff annotation files and properly indexed. Then, using the file as a reference gene file, HTSeq (v0.6.1p1) was used to estimate gene expression levels from the paired-end clean sequence data.

##### Differential expression analysis

Differential expression analysis was conducted using the DESeq Bioconductor package that incorporates a model based on a negative binomial distribution of sequence abundances. After adjustment using Benjamini and Hochberg’s approach for minimizing the false discovery rate, a *p* value of < 0.05 was used to detect differentially expressed genes.

##### GO and KEGG enrichment analysis

The GO-TermFinder program was used to identify gene ontology (GO) terms among the annotated list of enriched genes exhibiting significantly different expression levels. The Kyoto Encyclopedia of Genes and Genomes (KEGG) is a collection of databases incorporating genomes, biological pathways, diseases, drugs, and chemical substances (http://en.wikipedia.org/wiki/KEGG). In-house scripts were used to identify significantly differentially expressed genes among different KEGG pathways. Lastly, the novel transcript prediction program Rockhopper uses a Bayesian approach to construct a transcriptome map including transcription start/stop sites for protein-coding genes and novel transcripts and was used to construct such a map using the transcriptional data generated here.

## Additional files

**Additional file 1.** The method of synthesis of Mg-Fe-LDH NPs, preparation of Aodex/Mg-Fe-LDH, and adsorbent characterization. **Figure S1.** Characterization of the adsorption of Aodex by Mg-Fe-LDH NPs. **Table S1.** Genome annotation of *Arthrobacter oxidans* KQ11. **Table S2.** for summary of raw and filtered reads; and Illumina transcriptome reads mapped to the *A. oxidans* KQ11 genes. **Table S3.** for transcriptome annotation of *A. oxidans* KQ11 response to Mg-Fe-LDH NPs. **Table S4.** for significantly differential expressed genes of A. oxidans KQ11 with the Mg-Fe-LDH NPS treatment. **Table S5.** for the Shine-Dalgarno sequences of *A. oxidans* KQ11 with the Mg-Fe-LDH NPs treatment. **Table S6.** for sRNA of the new transcripts in the *A. oxidans* KQ11 intergenic region with Mg-Fe-LDH NPs treatment were annotated by NR.

## Data Availability

All data generated or analyzed during this study are included in this published article and its additional file.

## Abbreviations

NPs: nanoparticles
LDH NPs: layered double hydroxide nanoparticles
Mg–Fe-LDH NPs: Mg–Fe layered double hydroxide nanoparticles
*A. oxidans*: *Arthrobacter oxidans*
Aodex: *A. oxidans* KQ11 dextranase
*E. coli*: *Escherichia coli*
*B. subtilis*: *Bacillus subtilis*
GH: glycoside hydrolase families
DNS: 3,5-dinitrosalicylic acid
SEM: scanning electron microscopy
TEM: transmission electron microscope
PBS: phosphate-buffered saline
EDS: energy-dispersive spectroscopy
ICP-AES: inductively coupled plasma atomic emission spectrometer
GO: gene ontology
KEGG: Kyoto encyclopedia of genes and genomes
DEGs: differentially expressed genes
FPKM: fragments per kilobase of transcript sequence per millions base pairs sequenced
OD: optical density
